# Reply to Mucci, D.; Cioffi, B. Comment on “Al Barri et al. Evaluation of Refractive Predictive Accuracy in Intraocular Lens Power Calculations: A Comparative Study of Swept-Source Optical Coherence Tomography and Optical Low-Coherence Interferometry. *J. Clin. Med.* 2025, *14*, 1201”

**DOI:** 10.3390/jcm14228061

**Published:** 2025-11-14

**Authors:** Leila Al Barri, Nadina Mercea, Yasar Ionela-Iasmina, Mihnea Munteanu, Horia T. Stanca

**Affiliations:** 1 Ophthalmology Department, “Victor Babes” University of Medicine and Pharmacy, 300041 Timisoara, Romania; leila.albarri@umft.ro (L.A.B.); nadina.mercea@umft.ro (N.M.); ionela.yasar@umft.ro (Y.I.-I.); 2 Oftalmo Sensory-Tumor Research Center—ORL (EYE-ENT), Ophthalmology Department, “Victor Babes” University of Medicine and Pharmacy, 300041 Timisoara, Romania; 3 Ophthalmology Department, “Carol Davila” University of Medicine and Pharmacy, 050474 Bucharest, Romania; horia.stanca@umfcd.ro

Thank you for your thoughtful comments [1] on our article, “Evaluation of Refractive Predictive Accuracy in Intraocular Lens Power Calculations: A Comparative Study of Swept-Source Optical Coherence Tomography and Optical Low-Coherence Interferometry.” We appreciate your insights and will consider them for future research.

Regarding inter-eye correlation, while not explicitly corrected, its impact on results is generally considered minimal when refractive outcomes are similar. To address potential sample size disparity, we used bootstrapping, confirming the stability and robustness of our findings [2].

We conducted thorough normality assessments and applied appropriate statistical tests. Sensitivity analyses confirmed that our conclusions were not driven by sample size differences or the inclusion of both eyes. While Generalized Estimating Equations and mixed-effects modeling are valuable, our study’s design and research questions did not necessitate these more complex models for primary outcomes. Further sensitivity analysis confirmed outliers did not significantly affect overall results, and our power analysis minimized Type II error risk.

We acknowledge the limitations of a retrospective design; however, it provided valuable insights from real-world clinical data, reflecting typical practice and enhancing external validity despite non-randomized assignment.

Our results are robust and consistent with the existing literature, contributing meaningful evidence to IOL power prediction. We individually optimized lens constants for each device, focusing on the overall performance of the Barrett Universal II formula in a clinical setting.

We greatly appreciate your detailed review and look forward to incorporating these valuable suggestions into future analyses, continuing our dialog in advancing IOL power prediction.

## Data Availability

The data supporting the findings of this study are available from the corresponding author upon reasonable request.
